# The role of microRNAs in liver cancer progression

**DOI:** 10.1038/sj.bjc.6606010

**Published:** 2010-11-23

**Authors:** S Huang, X He

**Affiliations:** 1 State Key Laboratory of Oncogenes and Related Genes, Shanghai Cancer Institute, Shanghai Jiao Tong University School of Medicine, No.25/Ln.2200, Xie Tu Road, Shanghai 200032, China

**Keywords:** microRNA, liver cancer, cell cycle progression, apoptosis, metastasis

## Abstract

Primary liver cancer, predominantly consisting of hepatocellular carcinoma (HCC), is one of the most common and aggressive human malignancies worldwide. MicroRNAs (miRNAs) are a class of small non-coding RNAs that regulate gene expression post-transcriptionally. Emerging evidence indicates that miRNAs are often deregulated in HCC, and that some specific miRNAs are associated with the clinicopathological features of HCC. Recent work demonstrates that miRNAs have essential roles in HCC progression and directly contribute to cell proliferation, avoidance of apoptotic cell death, and metastasis of HCC by targeting a large number of critical protein-coding genes. The discovery of the aberrantly expressed miRNAs and their corresponding targets has opened a novel avenue to investigate the molecular mechanism of HCC progression and to develop potential therapeutics against HCC. In this review, we summarise current knowledge about the roles and validated targets of miRNAs in liver cancer progression.

Primary liver cancer mainly refers to hepatocellular carcinoma (HCC), cholangiocarcinoma, and hepatic angiosarcoma. As the third leading cause of death from cancer, HCC accounts for 85–90% of all primary liver cancers and ranks as the fifth most prevalent malignancy worldwide (Farazi and DePinho, 2006). Despite great advances in the treatment of the disease, relapse and metastasis are frequently observed in the clinic, and the 5-year survival rate remains quite low among patients with HCC. The development and progression of HCC is typical of a multistage process. The transformation begins in the liver tissue undergoing chronic hepatitis or cirrhosis caused by external stimuli (hepatitis B virus (HBV) or HCV infection, intake of aflatoxin B1, or alcohol abuse), progresses through a series of hyperplastic and dysplastic stages, and ultimately acquires the malignant phenotype with intrahepatic metastasis and distal dissemination (El-Serag and Rudolph, 2007). The progression is thought to involve the deregulation of genes that are critical to cellular processes such as cell cycle control, cell growth, apoptosis, and cell migration and spreading. In the past decades, studies have focused on investigating the genes and proteins underlying the development and progression of HCC (Aravalli *et al*, 2008). Recently, an increasing number of reports have described a new class of small regulatory RNA molecules termed microRNAs (miRNAs) that are implicated in HCC progression.

MicroRNAs are evolutionarily endogenous non-coding RNAs that have been identified as post-transcriptional regulators of gene expression (Bartel, 2004). The miRNAs mainly bind to the 3′ untranslated regions (UTRs) of target mRNAs, resulting in mRNA degradation or the blockade of mRNA translation. Computational analysis indicates that >30% of protein-coding genes may be directly modulated by miRNAs. Identification of miRNA targets is becoming urgent for our understanding of miRNA function. Increasing evidence shows that miRNAs have significant roles in diverse biological processes (He and Hannon, 2004). Meanwhile, deregulation of miRNAs has been observed in a wide range of human diseases, including cancer (Calin and Croce, 2006). In human cancer, miRNAs can function as oncogenes or tumour suppressor genes during tumour development and progression (Esquela-Kerscher and Slack, 2006).

Most recently miRNAs were found to be frequently deregulated in HCC, and some specific miRNAs were found to be associated with the clinicopathological features of HCC, such as metastasis, recurrence, and prognosis (reviews by Braconi and Patel, 2008; Ladeiro *et al*, 2008; Mott, 2009). Moreover, compelling evidence demonstrates that miRNAs have important roles in HCC progression and directly contribute to the cell proliferation, avoidance of apoptosis, and metastasis of HCC. Identifying the miRNAs and their targets that are essential for HCC progression may provide promising therapeutic opportunities. In this review, we discuss recent advances in our knowledge of miRNAs and validated targets in HCC progression (Table 1) and discuss potential future perspectives.

## Deregulated miRNAs and cell cycle progression of hcc cells

It is well demonstrated that a defect in cell cycle control is an essential step in the development and progression of human cancer. A number of oncoproteins and tumour suppressors involved in cell cycle regulation are often aberrant in HCC, thereby promoting HCC cell proliferation. Recent reports showed that some miRNAs can modulate the major proliferation pathways through interacting with critical cell cycle regulators such as cyclin–cyclin-dependent kinase enzyme (CDK) complexes, cell cycle inhibitors of the Cip/Kip family, the phosphoinositide 3-kinase (PI3K)/AKT/mammalian target of rapamycin (mTOR) signalling cascade, and other cell growth regulatory genes.

Cyclins are a family of proteins that control the cell cycle progression by activating CDKs. Both cyclins and CDKs, the positive regulators of the cell cycle, are found to be targeted by miRNAs in HCC. Cyclin D2 and cyclin E2 were validated as direct targets of miR-26a, which exhibits reduced expression in HCC (Kota *et al*, 2009). Expression of miR-26a induces HCC cell cycle arrest associated with direct targeting of these two cyclins. The mir-122, which accounts for 70% of the total liver miRNA population, was found to be frequently downregulated in HCCs and in all HCC-derived cell lines. Coulouarn *et al* (2009) identified the liver-enriched transcription factors HNF1A, HNF3A, and HNF3B as central regulatory molecules for loss of miR-122 in HCC. The miR-122 can suppress HCC cell growth by directly targeting cyclin G1 expression (Gramantieri *et al*, 2007). By modulating cyclin G1, miR-122 influences p53 protein stability, and transcriptional activity, thus decreasing the G2–M phase as well as reducing the invasive capability of HCC-derived cells (Fornari *et al*, 2009). Serum response factor (SRF) and insulin-like growth factor 1 receptor (Igf1R), which both promote tumourigenesis, have also been validated as targets of miR-122 (Bai *et al*, 2009). The miR-195, one of the miR-15/16/195 family members, was significantly reduced in HCC tissues and cell lines. The miR-195 suppresses tumourigenicity and blocks the G1–S transition by repressing Rb-E2F signalling through directly targeting multiple molecules, including cyclin D1, CDK6, and E2F3 (Xu *et al*, 2009). CDK6 was also shown to be targeted by miR-124, which was silenced through CpG methylation in HCC and induced cell cycle arrest at the G1–S checkpoint (Furuta *et al*, 2010). In addition, miR-124 can mediate HCC cell growth arrest by directly targeting vimentin, SET, and MYND domain containing 3, and IQ motif containing GTPase activating protein 1 (Furuta *et al*, 2010).

On the other hand, some oncogenic miRNAs may exert their functions through the inhibition of cyclin-dependent kinase inhibitors (CDKIs), such as the members of Cip/Kip family. The p21, a p53 target of the Cip/Kip family, is a direct target of miR-106b and miR-93 (Ivanovska *et al*, 2008), which are overexpressed in HCC and may have critical roles in cell proliferation by regulating the G1-to-S cell cycle transition. The p27, a second member of the Cip/Kip family with a relevant role as a tumour suppressor in human cancer, is mostly controlled at the post-transcriptional level. The miR-221 and miR-222 can function as oncogenes in HCC by binding to target sites in the 3′-UTR of p27 (le Sage *et al*, 2007). Intriguingly, miR-221 and miR-222 have been reported to directly interact with p57, another member of the Cip/Kip family (Fornari *et al*, 2008). By controlling these two CDKIs, miR-221 and miR-222 can promote HCC cell growth by increasing the number of cells in the S-phase.

Phosphoinositide 3-kinase, a lipid kinase that integrates different signals to balance survival and apoptosis, represents a major signalling pathway for cell proliferation. The activation of this pathway can increase the activity of AKT kinase, which can phosphorylate mTOR, thereby promoting cell growth. The PI3K/AKT pathway is controlled by the tumour suppressor lipid phosphatase PTEN. Recently, mTOR was identified as a target of miR-199a-3p which can block the G1–S transition and sensitise HCC cells to doxorubicin challenge (Fornari *et al*, 2010). In addition, the DNA damage-inducible transcript 4 (DDIT4), a modulator of the mTOR pathway, was also found to be a bona fide target of miR-221 and miR-222 (Pineau *et al*, 2010). Notably, PTEN is a direct target of miR-21, miR-221, and miR-222, all of which are frequently overexpressed in HCC (Meng *et al*, 2007; Garofalo *et al*, 2009). Thus, PTEN could be repressed by these miRNAs, which results in HCC cell survival through PI3K/AKT pathway activation.

In addition, the deregulated miRNAs can affect HCC cell proliferation through other important cell cycle regulators. Let-7g inhibits the proliferation of HCC cells by downregulation of c-Myc and upregulation of p16(INK4A) (Lan *et al*, 2010). The miR-1 that is methylated in HCC suppresses tumour cell growth by downregulating its oncogenic targets c-Met, FoxP1, and HDAC4 (Datta *et al*, 2008). Stathmin1, a functional target of miR-223 that is downregulated in HCC, is a key microtubule regulatory protein that controls microtubule dynamics, cellular proliferation, and the S-phase of the cell cycle (Wong *et al*, 2008). The miR-375 inhibits the proliferation and invasion of HCC cells by targeting Hippo-signalling effector YAP (Liu *et al*, 2010). The miR-18a is preferentially increased in female HCC and stimulates the proliferation of HCC cells through downregulating the *ESR1* gene, which encodes ER*α*, thus potentially blocking the protective effects of oestrogen and promoting the development of HCC in females (Liu *et al*, 2009).

## Deregulated miRNAs and apoptosis of HCC cells

Apoptosis is a major barrier that must be circumvented during malignant transformation and tumour progression. Tumour cells evolve to evade apoptosis so that they can escape from the surveillance system and survive in the tumour environment. Many of the signals that elicit apoptosis converge on the mitochondria, which respond to proapoptotic signals by releasing cytochrome *c*, a potent catalyst of apoptosis. The Bcl-2 family of proteins, whose members have either proapoptotic (Bim, Bmf, Bax, Bak, Bid) or antiapoptotic (Bcl-2, Bcl-W, Bcl-XL, Mcl-1) function, have important roles in governing mitochondrial death signalling. A growing body of evidence shows that miRNAs can help cancer cells to evade apoptosis by directly targeting the Bcl-2 family genes in HCC. The cellular mRNA and protein levels of Bcl-w were repressed by miR-122, which subsequently reduced cell viability and caspase-3 activation (Lin *et al*, 2008). The let-7 family of miRNAs inhibits Bcl-xL expression and potentiates sorafenib-induced apoptosis (Shimizu *et al*, 2010). The miR-101 may exert its proapoptotic function by targeting Mcl-1 (Su *et al*, 2009). Intriguingly, both Mcl-1 and Bcl-2 were direct targets of miR-29, and the mitochondrial pathway was activated in miR-29-promoted apoptosis (Xiong *et al*, 2010). Enhanced miR-29 expression can sensitise HCC cells to various apoptotic signals and suppress the tumourigenicity of HCC cells. All of these miRNAs are frequently downregulated in HCC, thus making HCC cells more resistant to apoptosis by upregulating the expression of antiapoptotic genes. On the other hand, Bmf, a proapoptotic BH3-only protein, is a target of miR-221 in hepatic carcinogenesis (Gramantieri *et al*, 2009). The miR-25 exerts an antiapoptotic effect by targeting and inhibiting Bim (Li *et al*, 2009c).

MicroRNAs can also regulate apoptotic cell death by targeting other apoptosis related genes. The miR-224, which is upregulated in HCC, sensitises cells to apoptosis by inhibiting apoptosis inhibitor-5 and increases cell proliferation (Wang *et al*, 2008). The miR-602 has an antiapoptotic role in HBV-related HCCs by inhibiting RASSF1A (Yang *et al*, 2010b). Restoration of miR-203 in HCC cell lines induces apoptosis by targeting ABCE1 (Furuta *et al*, 2010).

## Deregulated miRNAs and the invasion and metastasis of HCC cells

Invasion and metastasis, two of the most critical hallmarks of cancer, are the leading lethal factors for malignant cancer in general and HCC in particular. The long-term survival of HCC patients after curative resection is still plagued by the major obstacle of a high recurrence rate, which is mainly because of the spread of intrahepatic metastasis. Identification of metastatic factors and understanding the mechanisms underlying metastasis are important for the treatment of HCC. An increasing number of pro-metastatic miRNAs and anti-metastatic miRNAs are identified as upstream regulators of metastasis related genes and have a fundamental role in the invasion and metastasis of HCC cells.

### Pro-metastatic miRNAs and their targets in HCC

Meng *et al* (2007) first reported that aberrant expression of miR-21 can not only contribute to HCC growth, but also mediate HCC cell invasion by directly targeting PTEN. The miR-21 can alter focal adhesion kinase (FAK) phosphorylation and the expression of matrix metalloproteases MMP2 and MMP9, both downstream mediators of PTEN involved in cell migration and invasion. Recently, PTEN was also found to be the direct target of miR-221 and miR-222, which induce TRAIL resistance and enhance HCC cell migration (Garofalo *et al*, 2009). Besides PTEN, miR-221 and miR-222 also directly regulate the expression of the protein phosphatase 2A subunit B (PPP2R2A) and TIMP3 tumour suppressors, thus activating the AKT pathway and metallopeptidases to promote HCC cell invasion and metastasis (Garofalo *et al*, 2009; Wong *et al*, 2010). Interestingly, TIMP3 was also a functional target of miR-181b (Wang *et al*, 2010).The miR-181b is induced by TGF-*β*, and enhances MMP2 and MMP9 activity by modulating TIMP3 levels, thus promoting migration and invasion of HCC cells. This TGF-*β*/miR-181/TIMP3 axis might serve as an important complement to the TGF-*β*-mediated metastasis network. One report (Zhang *et al*, 2009b) also showed a novel miRNA (miR-143) mediated by nuclear factor *κ*B (NF-*κ*B) that promoted the metastasis of HBV-related HCC (HBV–HCC). Upregulation of miR-143 expression by NF-*κ*B in HBV–HCC promotes invasion and metastasis by repression of fibronectin type III domain containing 3B. Additionally, miR-17-5p, which is upregulated in HCC, promotes HCC cell invasion dependent on the activation of p38 mitogen-activated protein kinase and increased phosphorylation of heat shock protein 27 (Yang *et al*, 2010a).

Recently, miR-30d and miR-151, two frequently amplified miRNAs on chromosome 8q24, were found to be involved in HCC invasion and metastasis (Ding *et al*, 2010; Yao *et al*, 2010). The chromosomal region 8q24 is a common recurrent amplification region and is related to the metastatic properties in HCC. The miR-30d is frequently upregulated in HCC and its expression is associated with intrahepatic metastasis. High expression of miR-30d can enhance intrahepatic and distal pulmonary metastasis of HCC cells by repressing the direct and functional target G-*α*i2. The miR-151, which is often co-expressed with the host gene *FAK*, increases HCC cell migration and invasion by directly targeting RhoGDIA, a candidate metastasis suppressor in HCC, thus resulting in the activation of Rac1, Cdc42 and Rho GTPases. Moreover, miR-151 can function synergistically with FAK to enhance HCC cell motility and spreading.

### Anti-metastatic miRNAs and their targets in HCC

The miR-122 is significantly downregulated in liver cancers and suppresses HCC intrahepatic metastasis by regulation of a disintegrin and metalloprotease family proteins ADAM10 and ADAM17 (Bai *et al*, 2009; Tsai *et al*, 2009). let-7g was also shown to be present at significantly lower levels in metastatic HCCs and may suppress HCC metastasis by targeting type I collagen a2 (Ji *et al*, 2010). The hepatocyte growth factor (HGF)/c-Met signalling cascade is considered to be widely involved in the tumour metastatic process. The HGF interacts with c-Met receptor tyrosine kinase and leads to invasive growth by stimulating cell motility, invasion, and protection from apoptosis. The c-Met is frequently associated with the aggressive nature and the poor clinical outcomes of many tumours including HCC. The exact mechanism of upregulation of this gene in cancer remains poorly understood. Recently, the c-Met oncogene has been shown to be regulated by miR-1, miR-34a, miR-23b, and miR-199a-3p, all of which are downregulated in HCC. Silencing of miR-1 can not only inhibit HCC growth, but also mediate HCC cell invasion by downregulating c-Met (Datta *et al*, 2008). The miR-34a decreased c-Met-induced phosphorylation of extracellular signal-regulated kinases 1 and 2, and inhibited tumour cell migration and invasion (Li *et al*, 2009a). The miR-23b decreased the migration and proliferation abilities of HCC cells by downregulating c-Met and urokinase-type plasminogen activator, the latter of which is a critical functional downstream target of HGF/c-Met signalling (Salvi *et al*, 2009). The miR-199a-3p, decreased in HCC, induced G1-phase cell cycle arrest, and reduced invasive capability by targeting c-Met and mTOR (Fornari *et al*, 2010). The miR-101, a miRNA repressed in HCC, inhibits the expression of the FOS oncogene post-transcriptionally, thereby reducing HGF-induced cell invasion and migration (Li *et al*, 2009b).

## Conclusion and future perspectives

**Figure 1:**
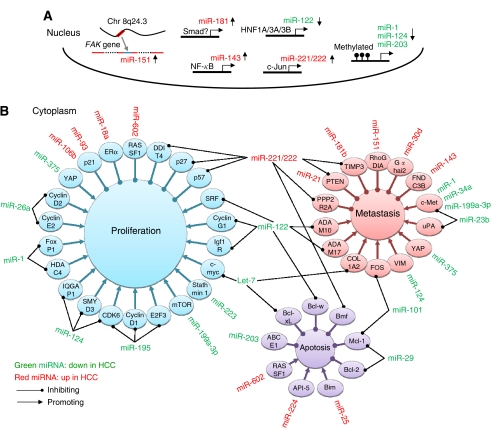
The interacting network of the aberrant miRNAs and their target genes in liver cancer progression. (**A**) The molecular mechanisms of the control of expression for some of the deregulated miRNAs. (**B**) The deregulated miRNAs and their target genes construct a complex interacting network to control HCC progression, and directly contribute to cell proliferation, avoidance of apoptotic cell death, and metastasis of HCC.

Current findings demonstrate that many miRNAs are differentially expressed in HCC, and have essential roles in liver cancer progression through directly targeting a large number of critical genes in HCC cells. Similar to protein coding genes, miRNAs are transcribed by RNA polymerase II and may be regulated at transcriptional levels. As shown in Figure 1A, miR-181 is regulated by TGF-*β* signalling; miR-143 is regulated by NF-*κ*B; miR-221 and miR-222 are regulated by HGF/c-Met signalling through the c-Jun transcription factor. Genetic and epigenetic aberrations may also contribute to the deregulation of miRNAs in HCC. For example, miR-1, miR-124, and miR-203 are silenced because of CpG methylation, and miR-151 is highly expressed in HCC because of the gain of chromosome 8q24. However, current knowledge about the molecular mechanisms of the control of expression for most of these deregulated miRNAs remains limited. Furthermore, the linkages between the aberrant miRNAs and the aetiology of HCC need to be further explored.

Although computational analysis indicates that one miRNA may directly modulate hundreds of mRNAs, such regulation has not been convincingly demonstrated experimentally. As shown in Figure 1B, some miRNAs have been shown to target multiple genes involved in HCC progression. For example, miR-221 and miR-222 can target *p27*, *p57*, *Bmf*, *PTEN*, *TIMP3*, *DDIT4*, and *PPP2R2A*; and miR-122 can target *cyclin G1*, *ADAM10*, *ADAM17*, *SRF*, *Igf1R*, and *Bcl-w*. As these target genes are involved in different cellular processes, individual miRNAs thus have multi-faceted roles in HCC progression. Conversely, a single gene may be regulated by multiple miRNAs. As shown in Figure 1B, c-Met, PTEN, TIMP3, Mcl-1, and CDK6 are found to be regulated by at least two miRNAs in HCC. Therefore, it is important to note that the alteration of the expression of a single miRNA may not be sufficient to influence a specific target gene. From these observations, we can deduce that the deregulated miRNAs and their target mRNAs construct a complex interacting network controlling HCC progression.

Technological advances indicate that the use of miRNAs or antagomir as therapeutics is feasible and safe. The discovery of aberrant miRNAs and their corresponding targets have already contributed to the development of miRNA-based therapeutics for HCC. Recently, Kota *et al* (2009) found that systemic administration of miR-26a in a mouse model results in inhibition of HCC cell proliferation, induction of tumour-specific apoptosis, and dramatic protection from disease progression without toxicity. This study provided an effective and promising strategy for future miRNA replacement therapies for the treatment of HCC. Note that these already identified deregulated miRNAs are aberrantly expressed and exert their functions only in a portion of HCC cases. One of the most important issues to be addressed is whether these deregulated miRNAs can be used to subtype HCC populations, categorising HCC cases into several subgroups based on their miRNA signatures, which will deepen our understanding of the underlying molecular mechanisms of hepatic carcinogenesis, and will facilitate the development of personalised miRNA-based therapeutics against HCC.

## Figures and Tables

**Table 1:** The aberrantly expressed miRNAs and validated targets in hepatocellular carcinoma

**miRNA**	**Expression**	**Regulation**	**Target genes**	**Function**	**References**
let-7	Down	ND	*c-Myc, Bcl-xL, COL1A2*	Grow (−), metastasis (−)	Ji *et al* (2010); Lan *et al* (2010); Shimizu *et al* (2010)
miR-1	Down	Methylation	*c-Met, FoxP1, HDAC4*	Grow (−), metastasis (−)	Datta *et al* (2008)
mir-17-5p	Up	ND	ND	Grow (+), metastasis (+)	Yang *et al* (2010a)
miR-101	Down	ND	*Mcl-1, FOS*	Grow (−), apoptosis (+), metastasis (−)	Su *et al* (2009); Li *et al* (2009b)
miR-106b-25	Up	ND	*p21, Bim*	Grow (+), apoptosis (−)	Li *et al* (2009c)
miR-122	Down	HNF1A, HNF3A, HNF3B	*Cyclin G1, SRF, Igf1R, Bcl-w, ADAM10, ADAM17*	Grow (−), metastasis (−), apoptosis (+)	Gramantieri *et al* (2007); Lin *et al* (2008); Bai *et al* (2009); Coulouarn *et al* (2009); Fornari *et al* (2009); Tsai *et al* (2009); Zhang *et al* (2009a); Ma *et al* (2010)
miR-124	Down	Methylation	*CDK6, VIM, SMYD3, IQGAP1*	Grow (−), metastasis (−)^a^	Furuta *et al* (2010)
miR-143	Up	NF-*κ*B	*FNDC3B*	Metastasis (+)	Zhang *et al* (2009b)
miR-151	Up	Gain on 8q24.3	*RhoGDIA*	Metastasis (+)	Ding *et al* (2010)
miR-181b	Up	TGF-*β*	*TIMP3*	Metastasis (+)	Wang *et al* (2010)
miR-18a	Up	ND	*ERα*	Grow (+)	Liu *et al* (2009)
miR-195	Down	ND	*cyclin D1, CDK6, E2F3*	Grow (−)	Xu *et al* (2009)
miR-199a-3p	Down	ND	*mTOR, c-Met*	Grow (−), metastasis (−)	Fornari *et al* (2010)
miR-203	Down	Methylation	*ABCE1*	Apoptosis (+)	Furuta *et al* (2010)
miR-21	Up	ND	*PTEN*	Grow (+), metastasis (+), apoptosis (−)	Meng *et al* (2007)
miR-221/222	Up	c-Jun	*p27, p57, DDIT4, PTEN, Bmf, TIMP3, PPP2R2A*	Grow (+), metastasis (+), apoptosis (−)	le Sage *et al* (2007); Fornari *et al* (2008); Garofalo *et al* (2009); Gramantieri *et al* (2009); Pineau *et al* (2010); Wong *et al* (2010)
miR-223	Down	ND	*Stathmin 1*	Grow (−)	Wong *et al* (2008)
miR-224	Up	ND	*API-5*	Grow (+), apoptosis (+)	Wang *et al* (2008)
miR-23b	Down	ND	*uPA, c-Met*	Metastasis (−)	Salvi *et al* (2009)
miR-26a	Down	ND	*Cyclin D2, Cyclin E2*	Grow (−)	Kota *et al* (2009)
miR-29	Down	ND	*Bcl-2, Mcl-1*	Apoptosis (+)	Xiong *et al* (2010)
miR-30d	Up	ND	*Gαi2*	Metastasis (+)	Yao *et al* (2010)
miR-34a	Down	ND	*c-Met*	Metastasis (−)	Li *et al* (2009a)
miR-375	Down	ND	*YAP*	Grow (−), metastasis (−)	Liu *et al* (2010)
miR-602	Up	ND	*RASSF1A*	Grow (+), apoptosis (−)	Yang *et al* (2010b)

Abbreviations: (−)=inhibition; (+)=promotion; down=downregulated; ND=not determined; up=upregulated.

a Hypothesis.
